# Compliant Interconnects Based on Single Micrometer-sized Metal-Coated Polymer Spheres

**DOI:** 10.1021/acsami.4c12039

**Published:** 2024-10-25

**Authors:** Van Long Huynh, Knut E. Aasmundtveit, Hoang-Vu Nguyen

**Affiliations:** Department of Microsystems, University of South-Eastern Norway, Raveien 215, 3184 Borre, Norway

**Keywords:** compliant interconnects, metal-coated polymer particles, particle deposition, nano Ag ink, low bonding temperature, low bonding pressure

## Abstract

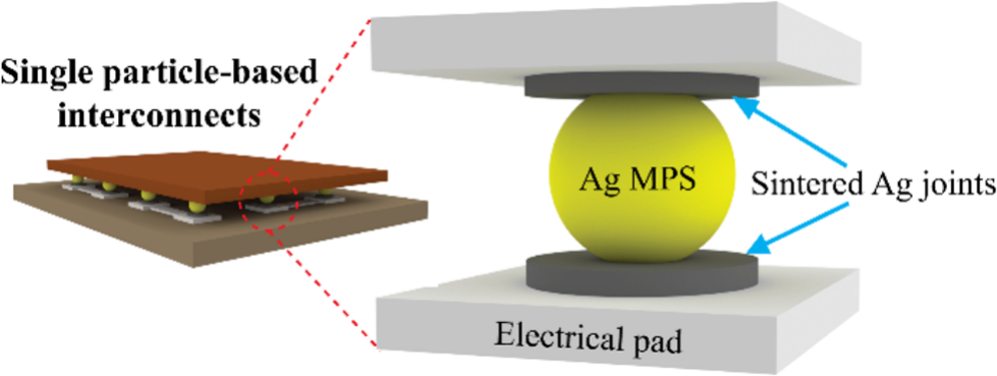

The rapid evolution of multifunctional electronics necessitates interconnection technologies appropriate for large dies with high-density and/or ultrafine pitch input/output pins. Existing technologies face numerous challenges, including demands for bonding equipment that can deliver extremely high force as well as thermo-mechanical stresses induced in the assembled packages due to mismatched thermal expansion of materials involved. This study proposes an approach to compliant interconnects comprising single micrometer-sized metal-coated polymer spheres, being joined to mating electrodes by sintering of Ag nano ink at low temperature (140 °C) and low pressure (∼15 mN/particle). Such an interconnection technology is expected to enhance the thermo-mechanical robustness of the assembled packages as well as be capable of high-density, ultrafine pitch interconnects. Our approach demonstrates control over conductive particles during assembly, achieving a 98% success rate in individual interconnects with a single captured particle. The use of sintered Ag not only secures free-standing particles on electrical pads (with an adhesion force above 2 μN) but also results in a 15% reduction in interconnect resistance, with measured resistance as low as 0.5 Ω, compared to interconnects without Ag ink. This method presents an alternative to metallurgical joints, particularly suited for high-density, ultrafine pitch applications, offering low bonding pressure and temperature, along with improved interconnect compliance to enhance the thermo-mechanical robustness of the packages.

## 1 Introduction

Electronic devices are continually shrinking in size while expanding in functionality, driving the demand for advanced electronic packaging and interconnection technologies capable of accommodating high input/output (I/O) counts with ultrafine pitch configurations. Since IBM introduced the Controlled Collapse Chip Connection (C4) technology in 1960 for flip-chip applications, which initially provided a maximum of 50 I/O counts per mm^2^ at a 150 μm pitch,^1^ numerous wafer bumping technologies have emerged, including micro solder bumps and copper pillars with solder caps. Micro solder bumps have become prevalent in flip-chip packaging.^2^ The recent development of the C4 Next-Generation Packaging (C4NP) process has enabled a significant increase in the I/O density to approximately 300 pin counts per mm^2^ at a 50 μm pitch.^3,4^ Alternatively, wafer-level sphere transfer processes, employing patterned vacuum tools to place micro solder balls, have demonstrated the potential to achieve even smaller pitches down to 30 μm.^5,6^ Copper pillar with solder caps has emerged as an alternative to conventional solder bumps, owing to its high aspect ratio, enabling higher density (up to 2500 I/O per mm^2^), finer pitch (20 μm), and improved thermal and electrical performance.^7^ It is worth noting that both micro solder bumps and copper pillars with solder caps offer self-alignment functionality during the reflow process.^1^ However, both technologies face a significant challenge: the coefficient of thermal expansion (CTE) mismatch among various materials during solder reflow, leading to thermo-mechanical stress within the assembled packages. This stress accelerates the formation of cracks in the packages.^7−9^ Numerous strategies have been explored to mitigate thermo-mechanical stress in the packages, including the use of low-melting-point Sn-based lead-free solders,^10^ like Sn–Ag, Sn–Cu, Sn–Bi, and Sn–Zn. Sn–Bi is notable for its low melting point of 139 °C.^11^ Research has shown that adding 5% In to Sn–Bi lowers the melting point to 119 °C,^12^ while a 51In-32.5 Bi-16.5Sn alloy melts at 59 °C.^13^ Though low melting points aid processing, they limit the application temperature range, and higher In content can reduce shear strength due to increased brittleness.^10,11^

The utilization of copper pillars with printed polymer core has been reported to effectively reduce thermo-mechanical stress in the package.^14,15^ A UV-cured polymer core as small as 20 μm in diameter and 25 μm in height is printed on the substrate by using aerosol jet printing, followed by Cu/Sn electroplating on the pillar core. The copper pillar with a polymer core exhibits 20% better die shear strength compared to conventional Cu pillars thanks to the excellent adhesion of the polymer core and SiO_2_ substrate. The elasticity of the polymer core reduces stress by 20% and increases characteristic life in temperature cycling and mechanical drop tests by 30% and 400%, respectively, compared with conventional solid copper pillars. However, the technique requires a series of complex processes, such as aerosol jet printing and metal deposition. These steps add to the complexity and potential cost of the manufacturing process. As an alternative approach to Cu pillars with printed polymer cores, metal-coated polymer spheres (MPS) have been utilized for fine-pitch, flexible, and stretchable interconnects where arranged MPS are embedded in a polymer matrix and integrated into the interconnection.^16,17^ This technology offers high-resolution interfacing (50 μm pitch) with low contact resistance (0.2 Ω per 0.25 mm^2^). However, to achieve a stable interface between the MPS and electrical pads, the assembly requires a hot-pressing process at elevated temperatures (235 °C) and high pressure (57 MPa).

In recent years, micro/nano Ag ink/paste, recognized as a low-temperature, low-pressure interconnection technology, has been widely adopted for flexible and stretchable electronics.^18,19^ These joints, commercially available in polymerizable and nonpolymerizable inks, offer a relatively simple process, relatively low sintering temperatures (160–200 °C), low sintering pressures (1–5 MPa), and low electrical resistances (sub-mΩ), positioning them as viable alternatives to Pb-free solder and Cu pillar bumps.^20^ Recent advancements in the dip-transfer process of Ag paste have enabled 50 μm pitch interconnects with electrical connectivity across over 700 connections.^19^ A significant challenge in Ag sintering is process optimization. A nonoptimized process can result in low electrical conductance and high porosity of sintered Ag joints.^21^

**Figure 1:**
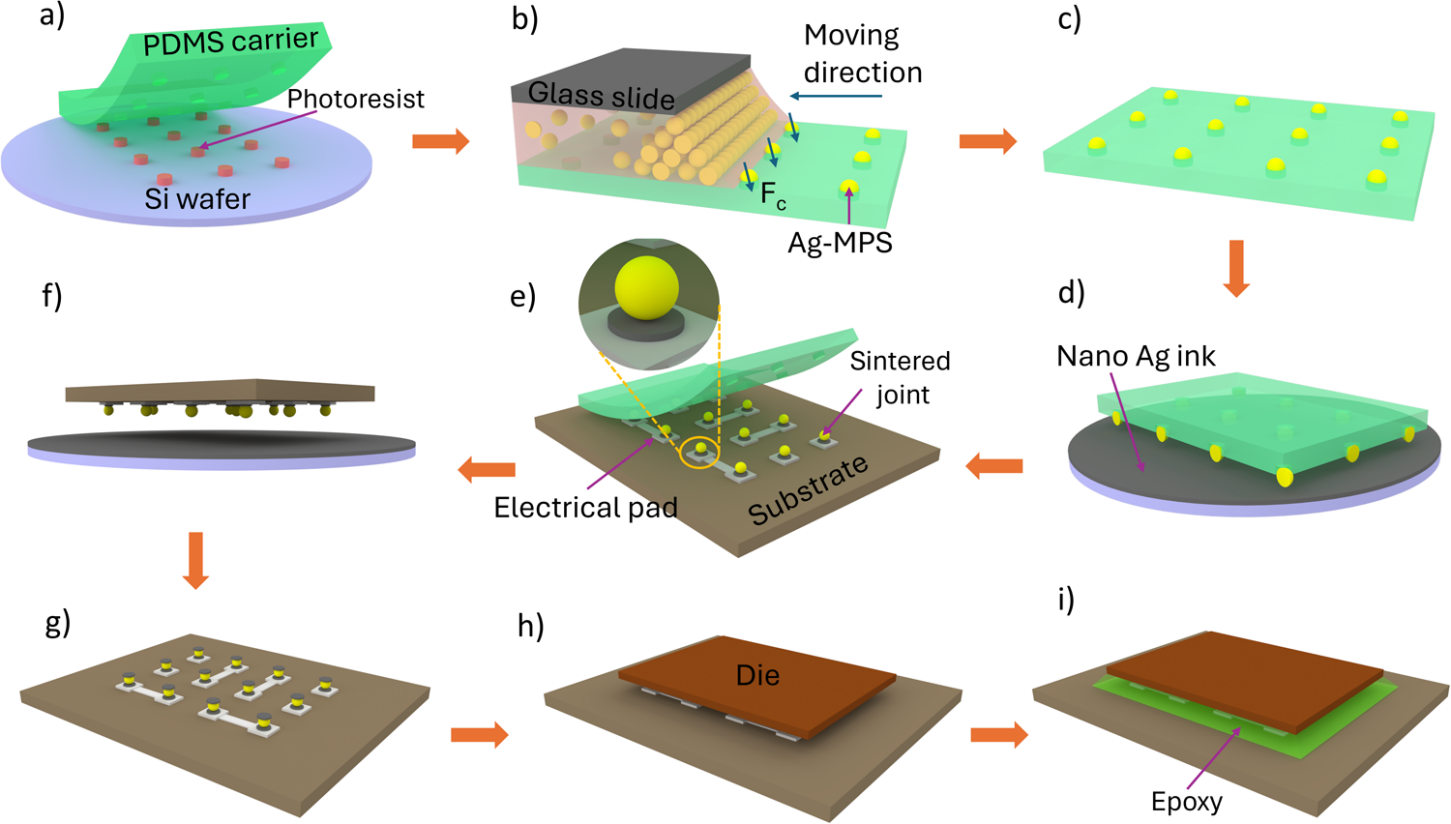
Assembly process of single-particle-based interconnects. (a) Fabrication of PDMS carrier using replication molding. (b, c) Capillary-assisted particle deposition onto predefined traps on the PDMS carrier. (d) Dipping the particles on the PDMS carrier into nano Ag ink. (e) Alignment and sintering process of conductive particles on substrate’s pads. (f) Second dip-coating of Ag ink onto the sintered particles on the substrate. (g) Flipping the substrate for (h) die alignment and a second sintering process. (i) Capillary underfilling and final curing process.

In this study, we present a new interconnection technology that combines Ag-coated polymer particles with low-temperature and low-pressure Ag sintering, aiming at enhanced compliance of high-density and ultrafine pitch interconnects. Our approach includes the use of nano Ag ink as a medium to facilitate the transfer of Ag-coated particles to electrical pads and further improve the electrical conductance of interconnects through metallurgical bonds between the Ag coating of particles and electrical pads by means of sintering the nano Ag ink at low temperature. Figure 1 illustrates our proposed methodology, which involves the selective deposition of MPS onto a patterned poly(dimethylsiloxane) (PDMS) carrier, followed by their transfer to the interconnects through two distinct Ag sintering processes. Our investigation focuses on the feasibility of the process, characterizing the behavior of conductive particles within sintered joints, and evaluating the electrical performance using samples with interconnection pitches of 175 and 200 μm.

## 2 Method

### 2.1 Design of Test Samples

**Figure 2:**
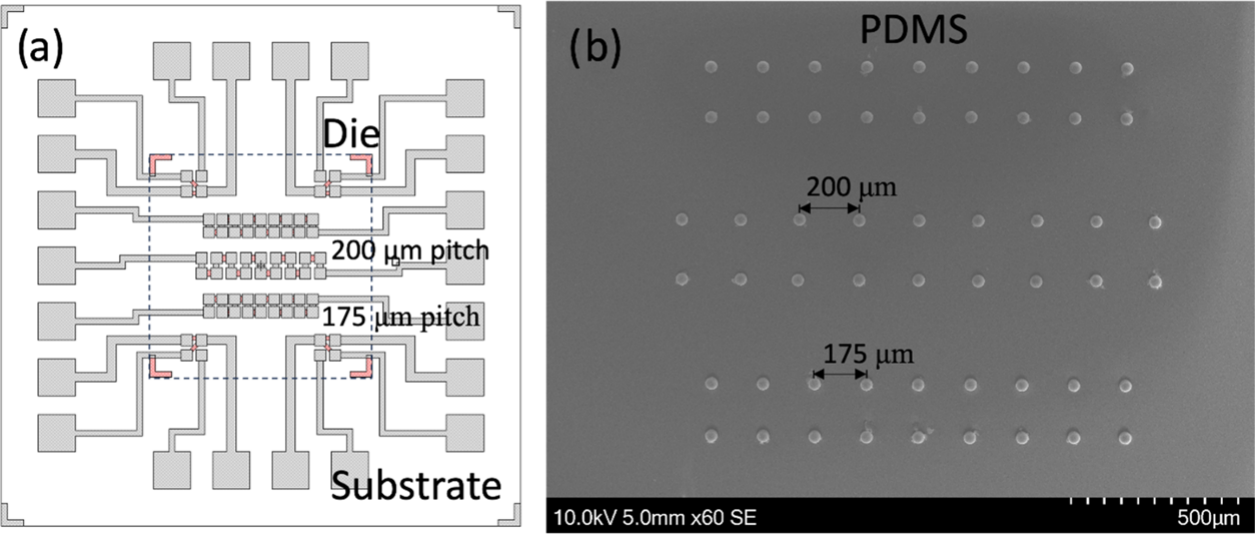
(a) Layout of test samples (a die overlaying a substrate) consisting of four four-point probe structures (200 μm pitch) at four corners and three daisy chain structures with two different pitches (175 and 200 μm) located at the center of the samples. (b) Deposition of conductive particles on predefined traps, where each deposited particle corresponds to an individual electrical pad on daisy chains on the substrate.

Test samples (Figure 2a) consisting of a Si substrate (7 × 7 mm^2^) and a glass die (3 × 3 mm^2^) were designed and fabricated with metallization layers of 10 nm Cr/100 nm Au on both die and substrate. Four four-point probe structures (200 μm pitch) were symmetrically patterned at the four corners, accompanied by three daisy chain structures (18 interconnects/each) at the center of the sample with two different pitches of 175 and 200 μm. The Cr/Au track resistance of both daisy chains with pitches of 175 and 200 μm was estimated to be 9.8 and 9.0 Ω, respectively, based on the geometry of the design and tabulated resistivity value of Cr/Au.^22^

### 2.2 Fabrication of PDMS Carrier and Particle Deposition

A master mold with an inverted design for replication molding of PDMS was fabricated through photopatterning of the SU-8 photoresist (Kayaku Advanced Materials). PDMS precursor (Sylgard 184 kit, Dow Corning) with a 10:1 ratio of prepolymer to curing agent was mixed and poured onto the master mold, followed by curing at 80 °C for 4 h. The cured PDMS carrier includes various trap holes, with each hole corresponding to an electrical pad on the substrate (Figure 1a).

Ag-coated polymer spheres, Ag-MPS (40 μm in diameter, Ag thickness of 120 nm, supplied by Conpart AS, Norway), were utilized as the conducting particles/media. Ag-MPS were dispersed in 0.01 wt % poly(ethylene glycol) tert-octylphenyl ether (Triton X-100, VWR Chemicals) in deionized (DI) water, yielding a final particle concentration of 2 wt %. The particle solution was sonicated immediately before the capillary assembly to improve uniformity and minimize the formation of particle clusters in the solution. A droplet of sonicated colloid particles was injected between the moving PDMS carrier and a stationary glass slide. Capillary forces induced particle deposition onto predefined traps when the meniscus of the droplet swept over the surface of the trap pattern (Figure 1b). The deposited particles on the PDMS carrier were dried at 70 °C for 5 min to remove any residual liquid inside the traps (Figure 1c).

### 2.3 Spin-Coating of Nano Ag Ink

Nano Ag ink (Silverjet DGP 40LT-15C, Advanced nano products, Korea) was spin-coated on a Si wafer at different spinning speeds of 1000, 1500, and 2500 rpm to obtain different ink thicknesses for particle dip-transfer. The thicknesses of the ink in the dry state were measured, allowing for the estimation of the corresponding wet ink. Assuming all of the solvents have evaporated in the sintered ink and the *X*–*Y* dimensions are the same in the dry and wet ink, the wet ink thickness is calculated based on
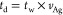
1

which gives
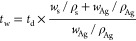
2where

- *v*_Ag_ is the volume fraction of Ag in the ink.
- *t*_w_ and *t*_d_ are the thicknesses of wet ink and dry ink, respectively.
- *w*_s_ and *w*_Ag_ are the weight fractions of solvent and Ag particles in the ink, respectively.
- ρ_s_ and ρ_Ag_ are the densities of solvent and pure Ag in the ink, respectively.

By using the tabulated values for triethylene glycol monomethyl ether solvent (*w*_s_ = 0.65, ρ_s_ = 1.026 g/cm^3^) and pure Ag (*w*_Ag_ = 0.35, ρ_Ag_ = 10.5 g/cm^3^),^22^ the thickness of wet ink is given by
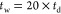
3

### 2.4 Sintering of Nano Ag Ink between Particles and Substrate

Ink deposition on particle and subsequent particle transfer was performed using a semiautomatic flip-chip bonder (FinePlacer Pico, Finetech GmbH, Germany), which facilitates the pick and place process with a placement accuracy of 5 μm and force applied between 1 and 40 N. Deposited particles on a PDMS carrier were brought into contact with Ag ink spin-coated on a Si wafer (Figure 1d). The PDMS carrier with particles was dipped in Ag ink and then aligned and firmly pressed to a substrate by applying a force of 1 N, followed by a sintering process at 140 °C for 2 min to facilitate the first Ag joints (Figure 1e). The parameters for Ag sintering were selected based on recommendations from the supplier.^23^ At the end of the sintering process, the PDMS carrier was carefully removed from the substrate, leaving free-standing conductive particles on the electrical pads.

### 2.5 Sintering of Nano Ag Ink between Particles and Die

Sintered Ag-MPS on the substrate were deposited with an additional Ag ink layer by repeating the dip-coating process (Figure 1f). The substrate was then flipped (Figure 1g), and a glass die was immediately picked and placed on top of the substrate, followed by the second sintering process at the same sintering temperature, 140 °C for 2 min (Figure 1h). During this process, a bonding force of 0, 1, or 2 N corresponding to 0 N, ∼15 mN, and ∼30 mN per particle, respectively, was applied. The assembly was allowed to cool down while maintaining the bonding force. Capillary underfilling (Epo-Tek 353, Epoxy Technology, USA) was introduced at one end of the die. The capillary force then drew the epoxy to the opposite end of the die, and epoxy curing was subsequently conducted at 140 °C for 2 min (Figure 1i). A total of 10 samples were prepared and characterized.

### 2.6 Transferring Deposited Particles from PDMS Carrier to Substrate Using Water-Mediated Layer

To study the impact of nano Ag ink on the transfer of particles to the substrate, deposited particles on the PDMS carrier were also transferred to electrical pads using a thin film of DI water/ethanol.^24,25^ A droplet of DI water/ethanol was spin-coated onto a substrate. The PDMS carrier with particles was brought into contact with the wet layer of DI water/ethanol, followed by the application of a 1 N force. The PDMS carrier was lifted off once the medium was fully evaporated at room temperature.

### 2.7 Visual and Electrical Characterization

Scanning electron microscopy (SEM, SU-3500, Hitachi, Japan) was employed to inspect particles in the deposition process as well as the cross-sectioning of bonded samples. The electrical resistance of bonded samples was measured by using a probe station (Micromanipulator, USA) integrated with a Keithley 2400 source meter (Keithley Instruments, USA). A profilometer (Dektak XT, Bruker, Germany) was employed to measure the thickness of the photoresist on the master mold. White-light interferometry (NT9100, Wyko, Germany) was employed to measure the thickness of the dry Ag ink that had been spun at various spinning speeds.

### 2.8 Bond Strength Characterization of Sintered Joints

Atomic force microscopy (AFM, XE-200, Park Systems, Korea) was employed to evaluate the adhesion strength of the sintered particles. Contact mode was utilized to map the position of particles on the electrical pads. To characterize the adhesion force of the sintered particles, force and distance microscopy was employed. This technique involved lifting the particle from the sintered joint by approaching and retracting an epoxy-coated AFM tip at specific positions on the particle. Calculating the maximum force that the lever can exert on the particles is crucial. The force corresponding to the maximum lever deflection was estimated as follows
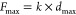
4where *k* is the spring constant, and *d*_max_ is the maximum nanometric vertical displacement of the lever, which gives
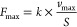
5

With *v*_max_ = 10 V of vertical deflection on the position-sensitive photodiode, sensitivity *S* = 50 V/μm, and *k* = 10 N/m (based on specifications of the AFM and cantilever), the maximum force at which the lever can pick up the particles is approximately 2 μN.

### 2.9 COMSOL Model of Particle Deformation and Electrical Resistance

A 2D model was constructed in COMSOL Multiphysics to simulate single Ag-MPS (with a diameter of 40 μm and Ag thickness of 120 nm) sandwiched between two Au pads, aiming to characterize the stress distribution on the Ag shell at a certain input deformation. The polymer core was modeled as a hyperplastic material, with assigned bulk modulus (*k*) and density (ρ) values of 104 MPa and 1100 kg/m^3^, respectively. The material properties for the Au pads were set as follows: Young’s modulus (*E*) = 700 MPa, density (ρ) = 19 300 kg/m^3^, and Poisson’s ratio (*ν*) = 0.44, as per the COMSOL material library. Similarly, default values from the library were adopted for the properties of the Ag coating layer: *E* = 83 × 10^3^ MPa, ρ = 10 500 kg/m^3^, and *ν* = 0.37. A fixed boundary condition was applied to the bottom Au pad, while a prescribed displacement from 0 to 25 μm with a displacement step of 1 μm was applied to the top Au pad, corresponding to particle deformation from 0 to 62.5%.

The resulting deformation models were utilized to estimate the resistance of individual deformed particles within the interconnect. Electrical conductivity values for the Au pad and Ag shell were set at 45.6 × 10^6^ S/m and 61.6 × 10^6^ S/m, respectively.^22^ Poly(methyl methacrylate) (PMMA) was chosen as the core material with an electrical conductivity of 10^–19^ S/m (COMSOL material library). A terminal current of 10 mA was applied to the top electrical pad, while the bottom pad was designated as ground.

## 3 Results

### 3.1 Fabrication of PDMS Carrier and Particle Deposition

PDMS carriers containing particle traps corresponding to the position of electrical pads on the substrate were successfully fabricated by using the replication molding method. The dimensions of the traps measured 50 μm in diameter and (20 ± 2) μm in depth (by measuring the thickness of photoresist features on the master mold). The deposition of conductive particles on the PDMS carrier reached a deposition yield of 100%. Figure 2b shows the deposition of Ag-MPS on predefined traps corresponding to three daisy chain structures on the substrate.

### 3.2 Sintering of Nano Ag Ink between Particles and Die/Substrate

**Figure 3:**
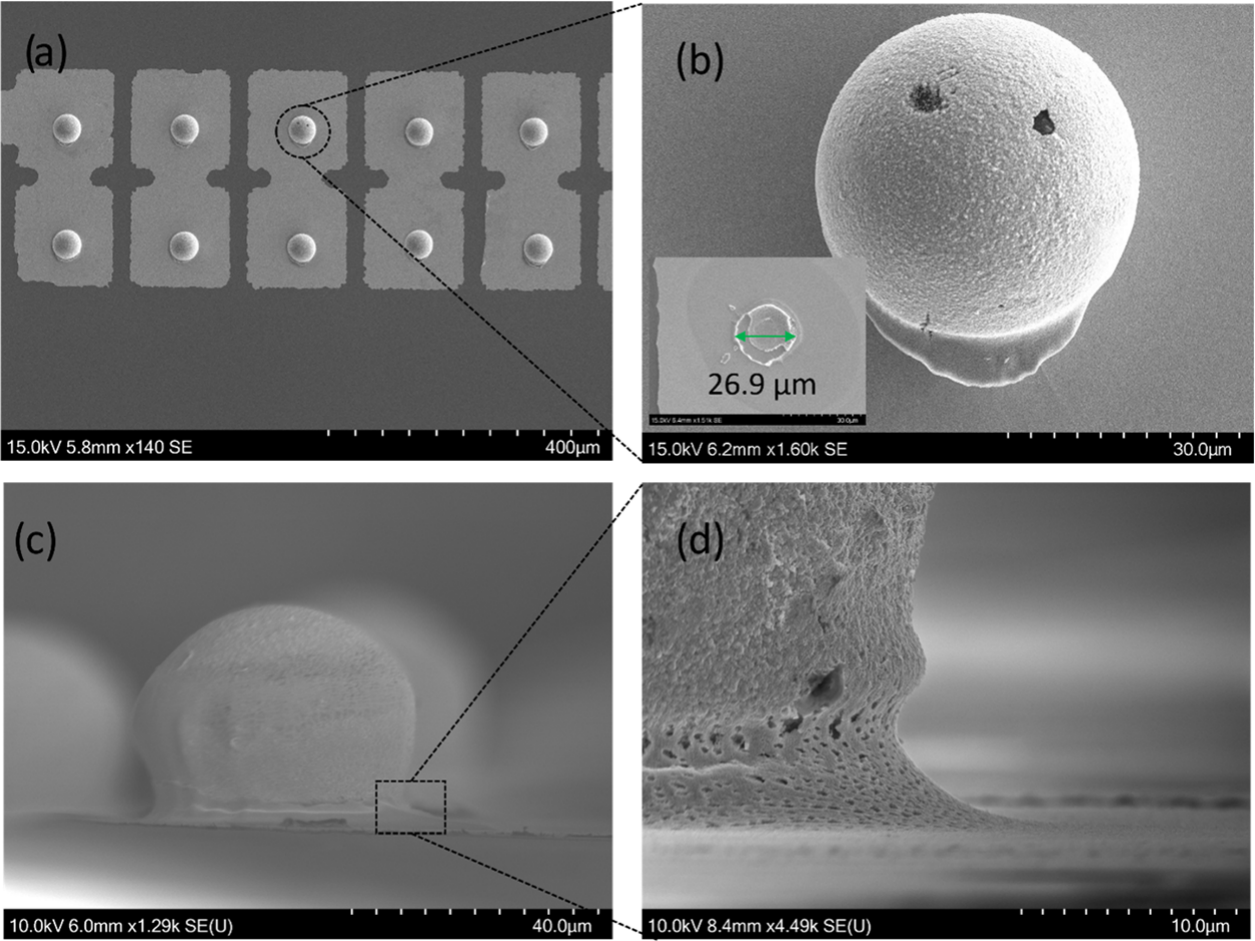
SEM micrographs of sintered particles on the substrate. (a) Transfer of conductive particles on electrical pads using Ag sintering with (b) higher magnification on a single pad, revealing a Ag layer between the particle and Au pad. (c) Side view of sintered particle with (d) higher magnification of the Ag neck.

Table 1 summarizes the measured ink thicknesses at different spinning speeds of 1000, 1500, and 2500 rpm. The transfer yield, as determined by counting the number of particles successfully transferred to the electrical pads relative to the total number of transferred particles, was observed to increase with increasing wet ink thickness. The highest transfer yield of 98% was achieved at a spinning speed of 1000 rpm corresponding to the wet ink thickness of ∼16 μm. Figure 3a,b depict the transfer of conductive particles onto the electrical pads after sintering at 140 °C, demonstrating the formation of a sintered Ag layer between the particles and the pads. A side view of the transferred particles (Figure 3c) provides a clearer visualization of the Ag neck between the particle and pad, showcasing its porous structure (Figure 3d). The dimensions of the sintered ink layer between the particles and pads were characterized by shearing off a particle, which yielded an ink diameter of (25 ± 5) μm in Figure 3b (inset).

**Table 1:** Estimation of Wet Ink Thickness versus Transferring Yield of Conductive Particles at Different Spinning Speeds

spin speed (rpm)	measured dry ink thickness (μm)	estimated wet ink thickness (μm)	transfer yield (%)
1000	0.78 ± 0.13	15.6 ± 2.6	98
1500	0.12 ± 0.04	2.4 ± 0.8	97
2500	0.09 ± 0.02	1.8 ± 0.4	91

### 3.3 Adhesion Strength of Sintered Particle

**Figure 4:**
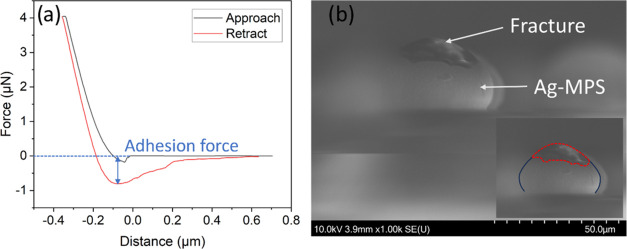
(a) AFM force/distance curve measured for sintered Ag-MPS on a substrate. (b) Side view of the particle after an attempt to pick up by using AFM tip revealing the broken Ag shell as well as deformation of the particle (inset schematic).

AFM characterization using force and distance microscopy showed that the AFM tip was unable to lift the particle out of the sintered joint. The adhesion force obtained from the typical force/distance curve was 0.9 ± 0.2 μN (Figure 4a). Microscopy of the characterized particle revealed a deformed particle with a broken Ag shell (Figure 4b).

### 3.4 Electrical Performance of Bonded Samples

**Figure 5:**
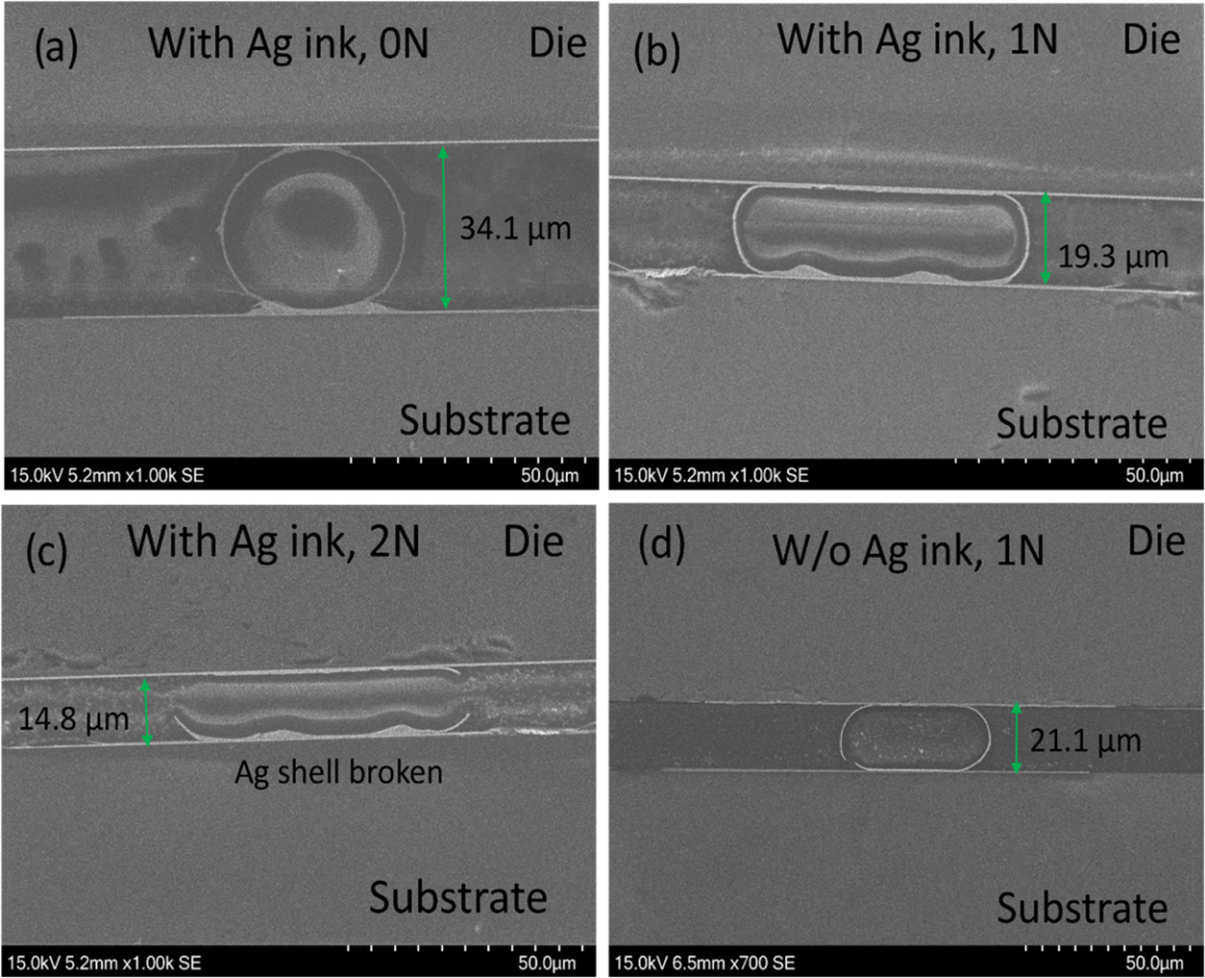
SEM micrographs of particle deformation with the presence of Ag ink when (a) no bonding force was applied, (b) 1 N was applied (∼15 mN/particle), and (c) 2 N was applied (∼30 mN/particle). (d) Interconnect without Ag ink at a bonding force of 1 N (∼15 mN/particle).

**Figure 6:**
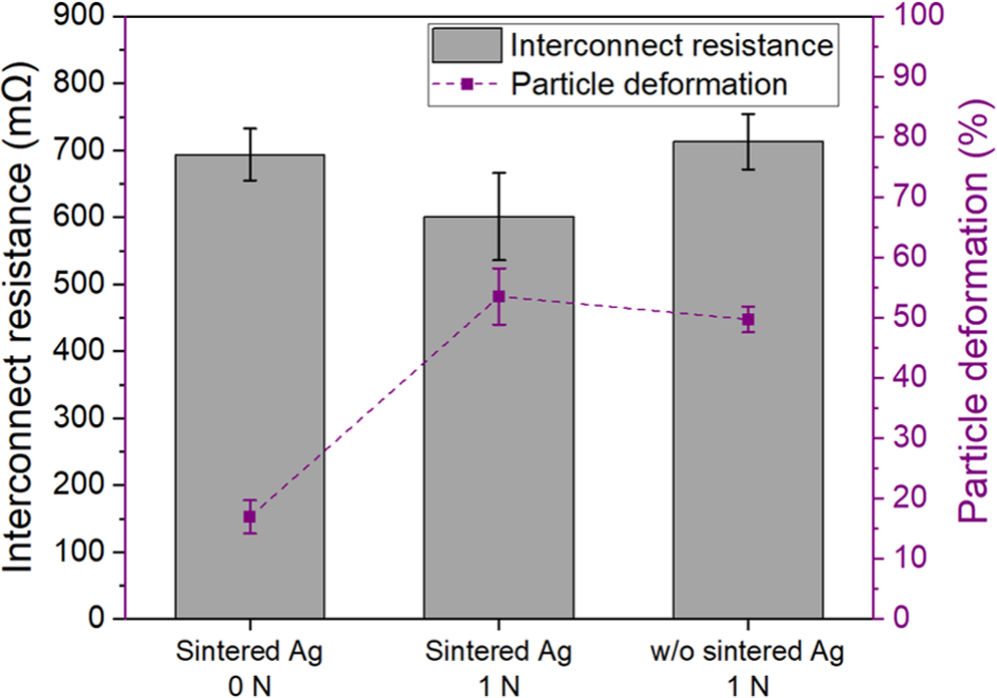
Interconnect resistance versus particle deformation of interconnects using Ag ink at different bonding conditions compared to interconnects without Ag ink. The presented data are from interconnects in four-point probe structures.

Table 2 presents the interconnect resistance measured using four-point probe structures, yielding values ranging from 0.5 to 0.7 Ω. Cross-section micrographs of bonded samples, taken from the measured four-point probe structures, are shown in Figure 5. Samples bonded without applying any bonding force exhibited an interconnect resistance of approximately 0.7 Ω (Figure 6), with particle deformation ranging from 14 to 19% (Figure 5a) compared to the original particle diameter. Applying a bonding force of 1 N reduced the interconnect resistance to approximately 0.6 Ω and increased particle deformation to 48–57% (in Figure 5b). Increasing the bonding force to 2 N resulted in open circuits, suggesting that the Ag coating layer on the particles was broken (Figure 5c). Interconnects fabricated without using Ag ink and with a bonding force of 1 N showed a resistance of approximately 0.7 Ω (Figure 6), with particle deformation ranging from 47 to 52% (Figure 5d).

**Table 2:** Electrical Resistance Measured from Four-Point Probe and Daisy Chain Structures for All Samples with Nano Ag Ink Sintered under Different Forces

		daisy chains			
		175 μm pitch		200 μm pitch	
characteristic	four-point probe	entire chain	interconnect	entire chain	interconnect
resistance [Ω]	0.6 ± 0.1	24.4 ± 1.2	0.8 ± 0.1	23.1 ± 1.4	0.8 ± 0.1

The electrical resistance of daisy chains with pitches of 200 and 175 μm was measured to be 23.1 and 24.4 Ω, respectively. Among the 30 daisy chains investigated, three exhibited open circuits due to missing particles.

### 3.5 COMSOL Model of Particle Deformation and Electrical Resistance

**Figure 7:**
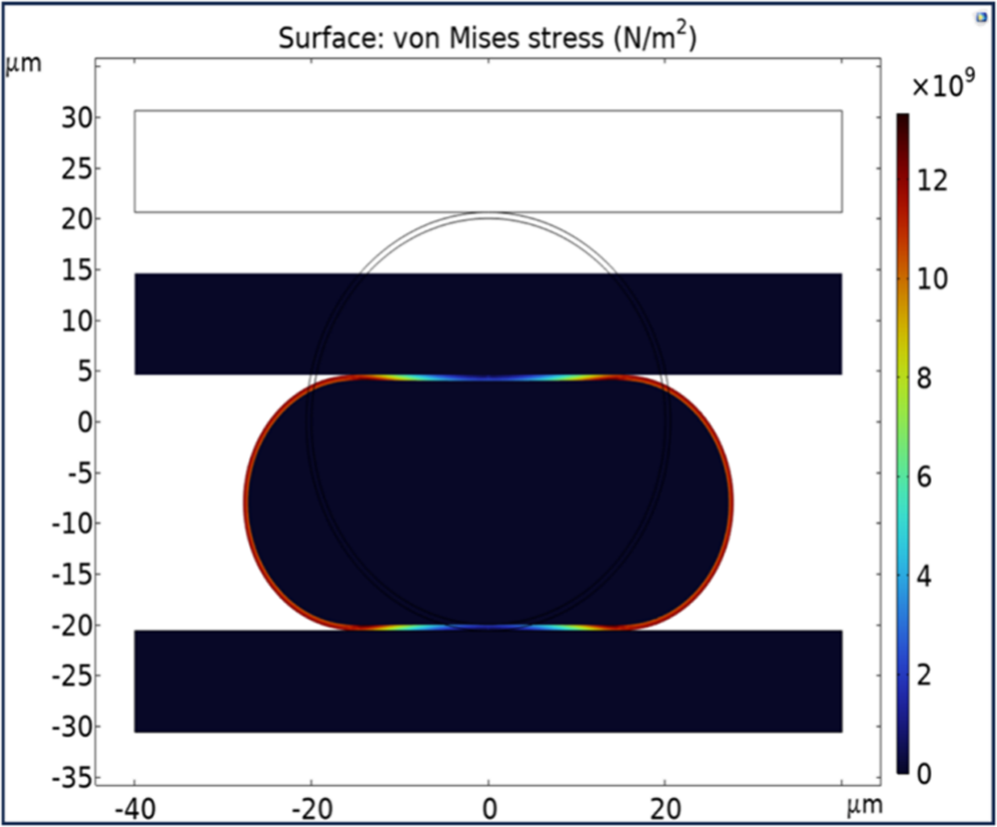
Von Mises stress of particle without Ag ink when deformed approximately 50% (correspond to Figure 5d).

**Figure 8:**
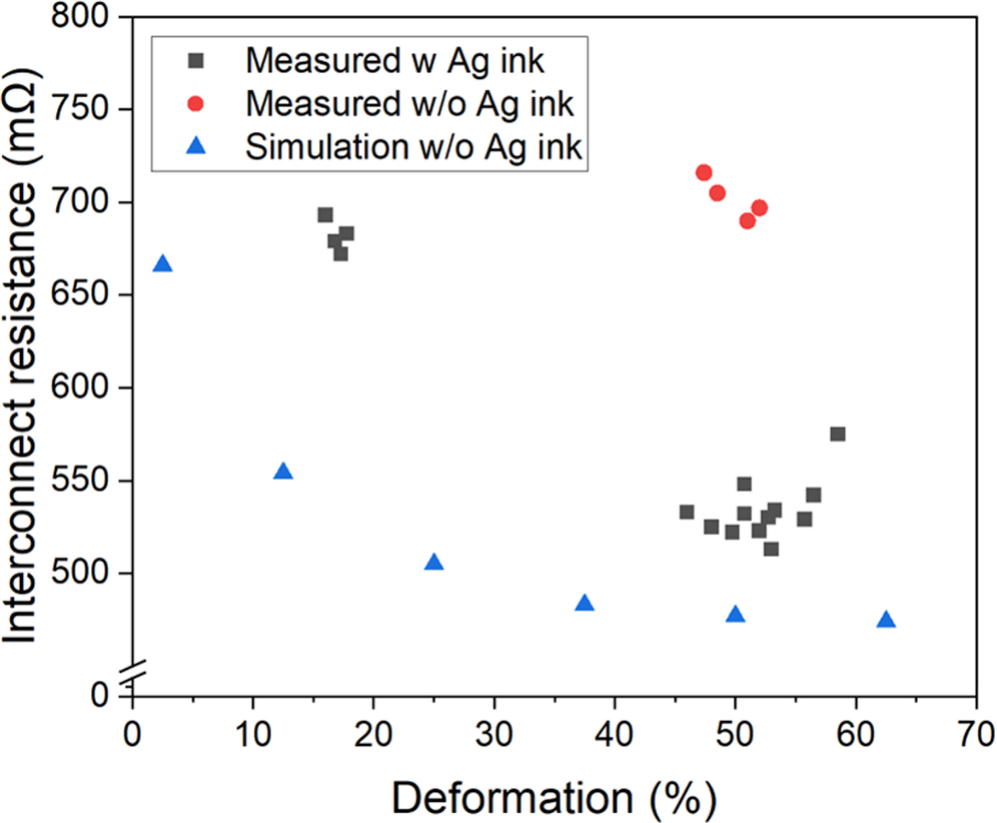
Interconnect resistance as a function of particle deformation of samples with and without Ag ink vs COMSOL simulation results for Ag-MPS particle.

Figure 7 displays a simulation of Von Mises stress distribution in a particle without nano Ag ink, showing a deformation of 21 μm (approximately 50% deformation), corresponding to Figure 5d. The simulation highlights a high-stress concentration at the rounded section, which corresponds to the broken Ag shell observed in Figure 5c when a larger bonding force is applied. Figure 8 presents the simulation results of electrical resistance as a function of particle deformation compared with measured resistances of samples with and without Ag ink. As observed, at the same degree of deformation, the COMSOL model without Ag ink exhibited lower electrical resistance, approximately 125 mΩ (∼50% reduction) and 50 mΩ (∼10% reduction) lower resistance compared to interconnects without Ag ink and with Ag ink, respectively.

## 4 Discussion

During capillary assembly, evaporation at the meniscus induces the formation of an accumulation zone due to long-range forces. As the meniscus moves over the trap surfaces, capillary forces deposit particles into the traps. To achieve a high deposition yield, it is essential to maintain the stability of the accumulation zone, which can be done by adjusting parameters, such as carrier temperature, deposition speed, trap dimensions, and the distance between traps.

The process of transferring conductive particles onto substrates using a Ag ink achieved a high transfer yield of 98%. The nanoscale size of the Ag particles, combined with the evaporation of the solvent at the sintering temperature, exposes large Ag surfaces, facilitating faster and more efficient sintering. It is important to note that the van der Waals attraction between the particles and the PDMS carrier,^26^ along with the low viscosity and surface tension of the Ag ink, ensures that the ink-deposited particles remain securely attached to the PDMS carrier for transferring. The shallow trap design (trap depth equal to half of the particle diameter of 40 μm) ensures a safe working distance (∼20 μm) between the PDMS carrier and the ink layer, preventing ink contamination of the PDMS carrier. Electrical pads with no particles transferred were seen, mainly near the edges of the substrates. The unsuccessful particle transfer is attributed to the uneven application of force during the transfer process. When a force was applied at the center of a PDMS carrier, it might cause a slight warpage on either side of the carrier. This resulted in nonuniform gaps between the PDMS carrier and the substrate along the carrier’s length, leading to no contact between a limited number of particles on the carrier and the substrate pads.

As seen in Figure 5, the interconnect displays the presence of a Ag neck between the particle and the Au pad in the absence of bonding force. When a bonding force is applied, the particle deforms, resulting in an overlap area between the particle and the Ag neck. In both bonding conditions, the absence of Ag necks between the particle and the Au pad on the top die was observed. This is due to the wettability of the nano Ag ink, which is drawn up along the particle surface when the substrate is flipped for die alignment. Ag necks between particles and electrical pads on the die could also be formed by reversing the process: first sintering particles on the die, then dipping the particles on the die into Ag ink, and performing a second Ag sintering on the substrate. Our approach demonstrates sensitivity to bonding pressure (below 15 mN per particle). Excessive bonding pressure can lead to the fracture of the metal shell(s), consequently resulting in open circuits.

We also attempted to transfer particles to electrical pads using a thin film of a water-mediated adhesive layer (Figure 5d). The resulting joint was weaker than sintered Ag joints,^24,25^ giving a lower transfer yield of 63%.

AFM characterization revealed that the AFM tip was unable to lift the particle at a maximum pick-up force of 2 μN. This indicates that either the adhesion force of the sintered particle exceeds 2 μN or the strength of the Ag shell is less than 2 μN, assuming sufficient contact between the AFM tip and the particle. Microscopy showed a broken Ag shell, and the force/distance curve indicated a measured adhesion force between the coated tip and the particle of 0.9 ± 0.2 μN. Our hypothesis is that the measured adhesion forces represent the force required to break the Ag shell. In other words, a single particle in the sintered joint exhibits an adhesion strength greater than 2 μN, corresponding to 3 mN/mm^2^ (calculated based on the maximum force that the lever can pick up and the dimensions of the sintered layer after shearing off, as shown in Figure 3b). It is expected that the adhesion strength of sintered joints would be relatively low due to the limited strength of the thin Ag shell compared to micro solder, Cu pillar, and Au stud bumps, which typically provide pull strengths of (30, 22, and 32) N/mm^2^ per interconnects,^27,28^ respectively. Therefore, underfilling is required to improve the mechanical strength of the package.

Figure 6 indicates that the measured interconnect resistance with Ag ink is slightly lower than that without the use of nano Ag ink when the same bonding force is applied. Sintered Ag not only assists in securing the particle on the electrical pad during the transferring process but also contributes to a reduction in interconnect resistance by approximately 15% (calculated from Figure 6) due to the formation of a metallurgical bond.

The simulation model of interconnects without nano Ag ink shows a significant drop in interconnect resistance with increasing particle deformation up to 40% (Figure 8). This is due to the combined effects of increasing the contact area between the particle and pad and reducing the distance between mating pads. Above 40% deformation, the resistance decreases slightly. This trend aligns with samples using Ag ink, where the resistance drops (∼150 mΩ) with increasing deformation. However, beyond 50% deformation, the measured resistance tends to increase due to high-stress accumulation in the Ag shell (Figure 7), leading to particle defects. In Figure 8, the COMSOL model without Ag ink shows lower electrical resistance, approximately 50 and 10% lower compared to interconnects without and with Ag ink, respectively. The difference in resistance could be attributed to crack formation between deformed particles and pads,^29,30^ the porosity of the Ag shell/ink, or contaminants.

The resistance of daisy chains with two different interconnect pitches exhibits a value similar to those obtained from the four-point probe measurements (Table 2). The slight differences observed may be attributed to uncertainties in estimating track resistance. The Cr and Au layers will interdiffuse, resulting in a higher resistivity than the tabulated values for Cr and Au, and uncertainties in the geometric values of the tracks will contribute.

The obtained interconnect resistance is much higher than other metallic joints, such as solder micro bumps,^31,32^ copper pillars with solder caps,^7,33^ and direct Cu–Cu thermocompression bonding,^34,35^ which typically exhibit resistances below 50 mΩ. It is worth noting that while metallic joints often require high bonding temperatures (typically above 200 °C) and/or high bonding pressures (above 2 MPa), the presented approach can be performed at a bonding temperature as low as 140 °C and a bonding force as low as 1 N (for a 3 × 3 mm^2^ chip with 70 interconnects). By utilizing COMSOL Multiphysics, it is conceivable to reduce the interconnect resistance by up to seven times (to approximately 90 mΩ) by increasing the thickness of the Ag coating layer by five times (to 600 nm). This modeling approach also facilitates the design of interconnects where the resistance can be predicted based on various inputs such as particle dimensions, deformation degree, coating materials, and coating thickness.

It is important to note that the samples used in this study are neither of high density nor of extreme pitch. However, it is feasible to increase the number of particle traps and decrease the distance between the nearest traps by appropriately designing the PDMS carrier. Based on our observations, the deposition yield will not decrease as long as the distance between two neighboring particles is greater than 1.5 times the particle diameter (60 μm). It is also possible to use particles as small as 4.8 μm in diameter for interconnects,^36^ with a pitch of approximately 7.5 μm. Thus, achieving high-density interconnects with pitches below 10 μm is potentially feasible.

## 5 Conclusions

Compliant interconnects consisting of single Ag-coated polymer particles (Ø40 μm, 120 nm thick Ag coating) have been demonstrated. The position of these particles within the interconnects is controlled by depositing them onto predefined traps on a PDMS carrier and subsequently transferring them to electrical pads by using Ag sintering. The particle deposition achieved a 100% yield, while the transfer yield of the particle reached 98%.

Sintering nano Ag ink at 140 °C forms Ag necks between the particles and the electrical pads on the substrate, facilitating particle transfer as well as reducing interconnect resistance. The adhesion strength of sintered particles is estimated to exceed 3 mN/mm^2^. The resulting interconnect resistance is ∼0.7 Ω with no bonding force applied and can be further reduced to ∼0.6 Ω by increasing the bonding force to 15 mN/particle. The utilization of nano Ag ink demonstrates a satisfactory transfer yield (98%) and a lower interconnect resistance (15%), compared to interconnects without Ag ink, attributed to the formation of metallurgical bonds.

The obtained results align with predictions from a model built by using COMSOL Multiphysics. The model has the potential to assist the design of single-particle interconnects in terms of suggesting particle types and relevant bonding pressures in accordance with specific requirements for interconnect resistance.
